# Optimizing Communication in Ataxia: A Multifaceted Approach to Alternative and Augmentative Communication (AAC)

**DOI:** 10.1007/s12311-024-01675-0

**Published:** 2024-03-07

**Authors:** Adam P. Vogel, Caroline Spencer, Katie Burke, Daniella de Bruyn, Peter Gibilisco, Scott Blackman, Jennifer M. Vojtech, Thayabaran Kathiresan

**Affiliations:** 1 Centre for Neuroscience of Speech, The University of Melbourne, 550 Swanston Street, Melbourne, VIC Australia 3010 Australia; 2 Redenlab Inc, Melbourne, Australia; 3 Department of Neurodegenerative Diseases, & Center for Neurology, Hertie Institute for Clinical Brain Research, University of Tübingen, University Hospital Tübingen, Tübingen, Germany; 4 Department of Speech, Language, and Hearing Sciences, Indiana University, Bloomington, USA; 5 Department of Speech and Language Therapy, Tallaght University Hospital, Dublin, Ireland; 6 Social and Political Sciences, The University of Melbourne, Melbourne, Australia; 7 Delsys, Inc, Natick, MA 01760 USA; 8 Department of Speech, Language, and Hearing Sciences, Boston University, Boston, MA 02215 USA

**Keywords:** Speech, Dysarthria, AAC, Communication, Ataxia, Language, Neurodegeneration

## Abstract

The progression of multisystem neurodegenerative diseases such as ataxia significantly impacts speech and communication, necessitating adaptive clinical care strategies. With the deterioration of speech, Alternative and Augmentative Communication (AAC) can play an ever increasing role in daily life for individuals with ataxia. This review describes the spectrum of AAC resources available, ranging from unaided gestures and sign language to high-tech solutions like speech-generating devices (SGDs) and eye-tracking technology. Despite the availability of various AAC tools, their efficacy is often compromised by the physical limitations inherent in ataxia, including upper limb ataxia and visual disturbances. Traditional speech-to-text algorithms and eye gaze technology face challenges in accuracy and efficiency due to the atypical speech and movement patterns associated with the disease.

In addressing these challenges, maintaining existing speech abilities through rehabilitation is prioritized, complemented by advances in digital therapeutics to provide home-based treatments. Simultaneously, projects incorporating AI driven solutions aim to enhance the intelligibility of dysarthric speech through improved speech-to-text accuracy.

This review discusses the complex needs assessment for AAC in ataxia, emphasizing the dynamic nature of the disease and the importance of regular reassessment to tailor communication strategies to the changing abilities of the individual. It also highlights the necessity of multidisciplinary involvement for effective AAC assessment and intervention. The future of AAC looks promising with developments in brain-computer interfaces and the potential of voice banking, although their application in ataxia requires further exploration.

Management of multisystem neurodegenerative disease necessitates that clinical care adapts to disease progression. In ataxia, speech, alongside other functional domains like upper and lower limb function often worsen over time [1–4]. Speech can deteriorate to the extent that verbal communication is no longer possible. When this happens, alternative and augmentative communication (AAC) approaches can help users meet their daily communication needs.

Traditional AAC methods are designed to assist users communicate via alternatives to natural speech. These can be low- or high-tech solutions and range from letter boards to button-activated voicing to eye tracking (see Table 1). These devices provide some capacity to communicate; however, the interaction is rarely as efficient or detailed as verbal communication.

**Table 1:** Current AAC resources

	Strengths	Limitations
Verbal AAC resources		
*Unaided:*		
Gesture	• Low tech • Low cost • Gesture is a natural part of communication already	• Gestures may be restricted to pointing; not sufficient for discussing abstract topics or objects • Not helpful for talking on the phone
Sign	• Low tech • Low cost (to implement) • Potential to enable the user to communicate in a more detailed/abstract manner	• Requires the speaker to learn the language • Requires a conversation partner to understand the signs used • Requires adequate upper limb dexterity
*Aided:*		
Communication book • Containing frequently used/functional phrases that are printed and can be shown to a communication partner	• Low tech • Low cost • Ability to communicate complex ideas clearly	• Requires carrying something extra • May be difficult to use/coordinate while maintaining balance (for ambulatory persons) • Requires manual manipulation
Life story book • Containing a written description of important events in the person’s life, including likes/dislikes, stories about family/partner/friends, medical diagnosis	• Low tech • Low cost • Ability to communicate important aspects of speaker	• Additional equipment • May be difficult to use/coordinate while maintaining balance (for ambulatory persons) • Requires manual manipulation
‘Pocket card’ outlining nature and cause of ataxia/dysarthria	• Low tech • Low cost • Ability to quickly educate an unfamiliar conversation partner about dysarthria and need for more time to communicate	• Doesn’t specifically aid speech
Pen and paper	• Low tech • Low cost • Potential to aid speech	• May not be suitable for people with poor dexterity or vision
Alphabet chart	• Low tech • Low cost • Can help with pacing speech as well as aid the listener (e.g. speaker points to the first letter of the target word)	• Requires carrying additional equipment • May be difficult to use/coordinate while maintaining balance (for ambulatory persons) • Requires manual manipulation • May not be suitable for a person with vision impairment
Pictures • Picture Exchange Communication System (PECS) • Photo album of important people or life events	• Low tech • Low cost • Potential to easily communicate with a familiar or unfamiliar communication partner	• Additional equipment • May be difficult to use/coordinate while maintaining balance (for ambulatory persons) • Requires manual manipulation • May not be suitable for a person with vision impairment
Message/voice banking • Voice memo app iPhone/iPad • ‘legacy recordings”	• A person can tell a story or a message in their own voice	• Historically needed to be completed before speech was significantly affected
Speech Generating Devices • Standalone device • iPad + app - e.g. GridPlayer, Proloquo2Go, Text to Speech!, Speak4Me, yes or no communication, LetMeTalk, Predictable • Eye gaze tracking • Switches (activated by head/finger/elbow, etc.)	• dedicated speech device • more widely available • more flexible as communication needs change (listening/speaking reading environments or as disease progresses) • may help compensate for hand motor difficulty • may help compensate for hand motor difficulty	• high tech • high cost • apps may have limited functions • may not be appropriate for patients with nystagmus • may not be appropriate for patients with significant limb discoordination
Closed Captions (for phone calls, video calls, educational videos, TV shows, movies, etc.)	• Commonly available on TVs, mobile phones, tablets, computers, social media videos	• Aids hearing, not speech • Not applicable in face-to-face conversations
Microphone	• Commonly available • May help increase intelligibility in persons who have difficulty maintaining phonation and respiratory support	• May not be helpful for more severe dysarthria • Requires additional equipment / speaker
Written communication AAC		
Voice-to-text dictation apps and software	• May compensate for fine motor difficulty	• Speech recognition software has difficulty deciphering dysarthric speech
Predictive text	• May help compensate for fine motor difficulty	• Still requires some fine motor performance
Screenreaders and apps	• May help compensate for visual difficulty	• Not all written material can be easily read

In multisystem conditions like Friedreich ataxia, use of AAC is restricted by physical limitations including upper limb ataxia and visual disturbance [5]. These deficits make it challenging to efficiently use or see a cursor on screen, or manipulate buttons on a device (e.g., phone/tablet). Speech-to-text algorithms have limited capacity to recognise unclear target words, eye gaze technology has difficulty accurately tracking jerky eye movements and all approaches require training, patience, content expertise from clinicians and are fatiguing for the patient. These limitations do not mean AAC is ineffective. They do, however, dictate that some devices are useful at specific disease stages, with their utility changing when symptoms worsen.

This perennial issue is currently managed from several directions. Firstly, maintaining existing speech abilities is paramount. Speech rehabilitation remains the mainstay of clinical practice. Advances in digital therapeutics has brought treatment into the home, providing greater access to therapies for patients [6]. Secondly, efforts are underway to improve the accuracy of speech to text for dysarthric speech. Google’s Euphonia project [7], for example, has an aim to *translate* unclear speech into intelligible communication.

Despite these options, there is limited knowledge and evidence on AAC use in ataxia. This review describes AAC resources, assessment practices in AAC, and how management can change with disease state. We also discuss new possibilities for optimising and maintaining communication in people with ataxia where verbal speech is not viable.

## Communication in ataxia

Spoken language is the primary form of communication for most people with ataxia. This is despite speech deteriorating as disease progresses. Dysarthria in ataxia is characterised by impairments across multiple speech subsystems, including respiration [8], phonation [9], articulation [10,11], resonance [12] and prosody [13]. These initially manifest as slurred and slower speech, gradually leading to a more severe phenotype, with vowel distortion, imprecise consonants, mixed nasality (predominantly hypernasality), reduced rate, and dysphonia. These combine to reduce intelligibility and naturalness of speech [14]. People with ataxia also present with difficulties across modes of communication, including hearing [15], vision [16] and writing/typing and reading impacted by upper limb impairment [17].

### AAC

AAC encompasses a wide range of modalities and tools designed to assist individuals with communication difficulties. These modalities can be categorized into unaided and aided communication. Unaided communication relies on the communicator's body and doesn't involve external tools or devices. This typically takes the form of sign language, gestures, facial expressions, and body language. Unaided communication is suited to individuals who have preserved motor control but cannot rely on verbal communication [18].

Aided communication requires external tools or aids to supplement or replace speech. It includes low- and high-tech solutions. Low-tech AAC includes basic tools like communication boards, paper-based systems, and symbol books. They are often cost-effective and easy to implement. High-tech AAC encompasses advanced options like tablet apps, dedicated communication devices, specialised digital hardware, speech generating devices (SGD), and computer software. These often offer greater versatility and can sometimes be customized to suit an individual's unique needs.

SGDs are electronic devices that produce speech based on user input. They are often used by individuals with more complex communication needs, including ataxia. These devices may use text-to-speech technology, pre-programmed messages, and predictive text options. Some SGDs can be controlled through physical movements, such as head movements or eye gaze, making them accessible to individuals with severe motor impairments [19].

## Assessment of complex communication needs

**Fig. 1:**
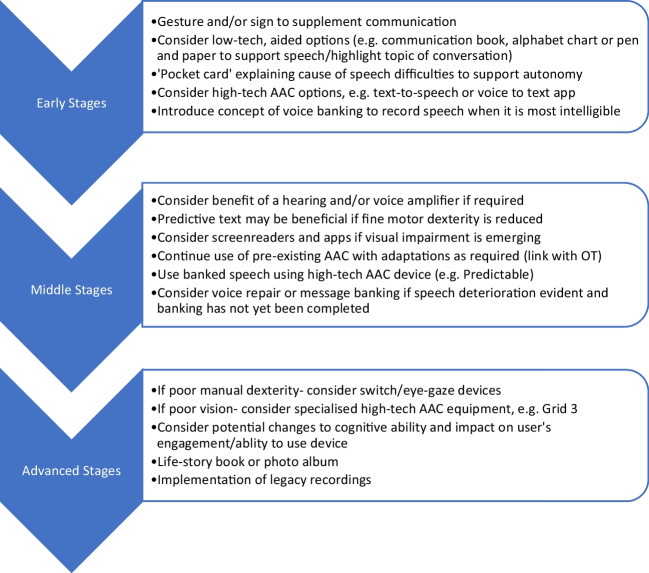
Changing AAC suitability across disease stages in ataxia

It is estimated that up to 0.5–1% of the world population may benefit from AAC, as they are unable to meet their daily communication needs through natural speech [20,21]. Complex communication needs manifest across a spectrum of disease severity and duration in ataxia, yielding diverse effects on an individual's engagement in domestic, professional, and other community settings. The dynamic nature of progressive ataxia warrants regular assessment of communication (see Fig. 1). Comprehensive testing is required to identify communication needs and the potential role of augmentative methods for supplementing non-functional speech [19]. There are no specific assessment procedures for evaluating communication needs in individuals with progressive ataxia [22]; instead, clinicians must establish a comprehensive picture of sensorimotor control and function alongside user preferences and participation needs.

AAC assessment and subsequent intervention typically requires active involvement from a team of individuals, including the person who requires AAC; their family members, caregivers, and communication partner(s); and clinical specialists [23]. Families, caregivers, and communication partners (e.g., teachers, employers, co-workers) play a key role in AAC assessments since—beyond the candidates themselves—this group of individuals is most likely to be affected by any decisions made toward an AAC intervention. The lack of family involvement in the assessment and intervention process has been cited as a significant factor in AAC device abandonment [24,25]. The involvement of a multidisciplinary team of specialists is crucial to identify an intervention that addresses the candidate’s unique communication challenges, which can include psychophysiological (e.g., sensory-perceptual, motor, cognitive-linguistic, and literacy impairments) and environmental considerations. The team of clinical specialists can therefore include professionals such as speech-language pathologists, physical therapists, occupational therapists, social workers, vocational coaches, medical personnel (e.g., audiologist, primary care physician), and psychologists, among other professionals. Together, this team is responsible for performing AAC assessments and, in turn, using assessment results to implement and modify AAC interventions as needed.

**Fig. 2:**
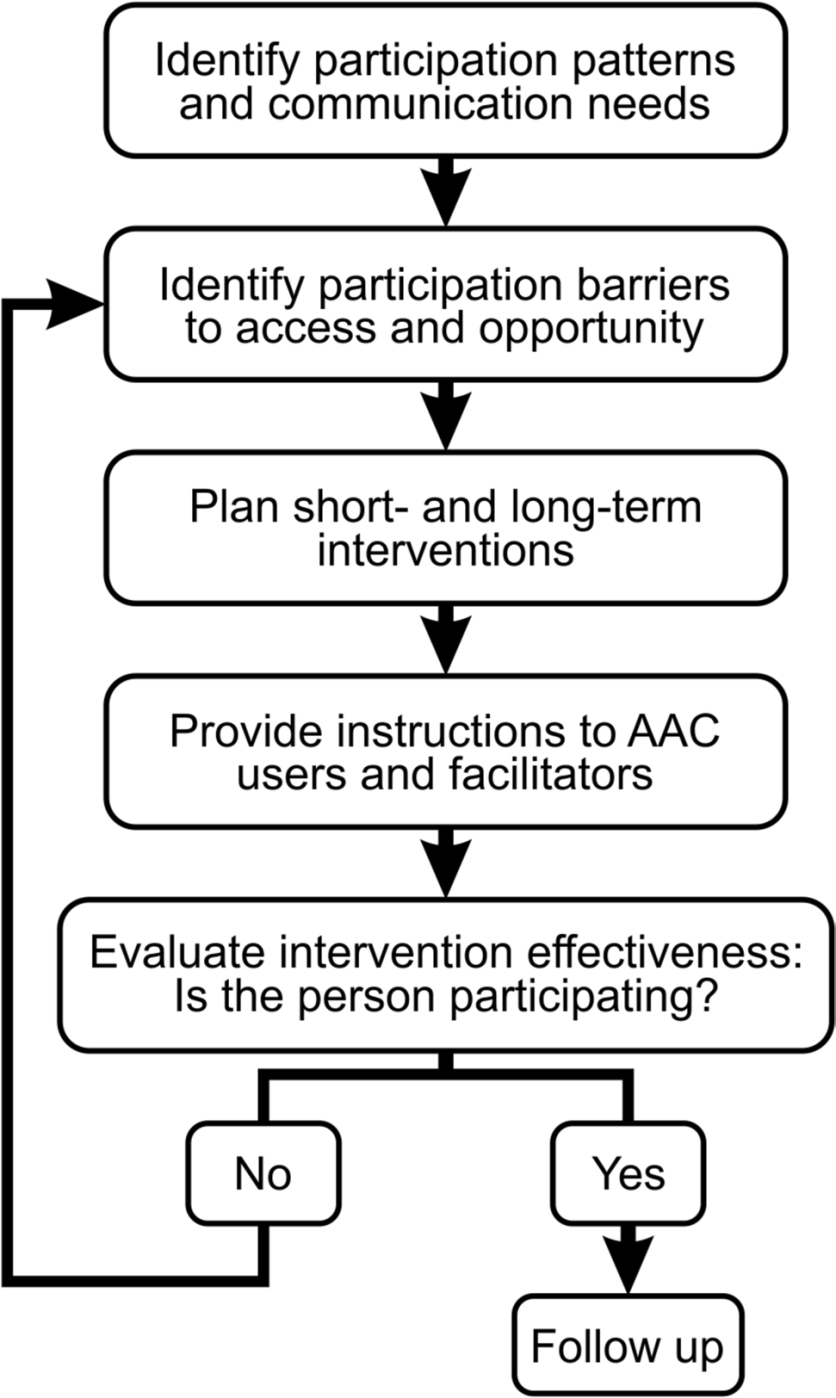
Schematized framework for assessing communication in ataxia, based on the revised Participation Model from Beukelman and Mirenda (2014)

AAC intervention is recommended by the American Speech-Language-Hearing Association (ASHA)^26^ to be considered as early as possible, regardless of communication impairment aetiology. Beukelman and Mirenda developed the Participation Model in 1988 as a framework to systematically implement AAC assessments; the model has since been revised in 2014. The revised Participation Model is recommended by the ASHA to guide AAC assessment and intervention as a dynamic process that needs to be revisited as AAC users learn new skills or as their disabilities progress (i.e., a change in “participation level”). According to the Participation Model, the focus of an AAC assessment is to gather information about the candidate’s participation patterns and needs, and then to plan, implement and evaluate the effectiveness of AAC interventions. Progress is then monitored and re-evaluated as needed based on how the user participates in their environments (see Fig. 2).

A comprehensive assessment includes collecting a case history, identifying communication skills and needs, characterizing facilitators and barriers, and assessing sensorimotor abilities (e.g., hearing, vision, motor). The goal of testing is to identify factors that contribute to communication success for the individual who relies on AAC as well as for their communication partners. AAC assessments should ideally take place over multiple days to gain clear insight into how an individual’s environment (e.g., lighting, device positioning, noise, familiarity, communication partner) and personal behaviours (e.g., attention, fatigue, motivation) affects their needs or skills.

Interviewing the candidate and exploring their social network are both valuable methods to delineate the candidate’s life experiences, goals, capabilities and strengths, limitations, and fears, and—most importantly—their unmet communication needs. This process can be used to highlight the candidate’s cultural, socio-economic, and linguistic background, as well as contextual factors (both personal and environmental), structural or functional impairments, comorbid deficits, and perspectives toward AAC. One popular instrument called the Social Networks Inventory is useful for characterizing interpersonal relationships, specifically in how the user feels about their relationships with significant others in their life and how they think those others feel about that relationship [27]. Speech and language assessments should be also conducted to comprehensively assess the candidate’s natural speech, expressive and receptive language, reading and writing abilities, symbol representation, and cognitive-linguistic organization. This largely includes informal assessments via interviews and observations with the user, but are often supplemented by standardized assessments such as the Comprehensive Aphasia Test [28](word- and sentence-level understanding), or the Peabody Picture Vocabulary Test–Fifth Edition (PPVT-5) [29] for receptive language.

Sensorimotor capabilities can be evaluated in areas of motor control, vision, and hearing. Motor control can be assessed by observing reflex patterns that may interfere with volitional control and characterizing the candidate’s ability to make gestures, manual signs, or dynamic movements. The latter is important when considering the ability of a person with ataxia to access aided AAC—such as using a finger to reliably select letters on a touchscreen—as they may have significant difficulty quickly and accurately controlling hand movements. Aided AAC modalities can be accessed by direct selection or scanning. Direct selection may involve pressing real or virtual buttons using different body parts (e.g., head, hands, fingers), or through hi-tech modalities such as head tracking, gaze selection, or eye tracking. If an individual has difficulty directly selecting items, then scanning may be used as an alternative; here, the AAC team must consider possible motor control sites as well as switch types that are used to make selections one at a time. This involves a process of trial and error, where users try a range of switches, movements, and positions to identify a combination that provides the most successful access to an activity. It is important to consider observe user position, movement patterns and concerns, control sites, type and positioning of switches, user skill levels, and the user’s preferred choice(s). Though primarily an informal and person-centered process, various frameworks exist to guide the AAC team with the process of a switch assessment. For instance, the ACE Centre in the UK developed the *Switch Assessment and Planning Framework for Individuals with Physical Disabilities* [30] to guide AAC teams in establishing a switch access method that offers the easiest, quickest, and most successful method of access. Observations from motor control assessments are used to identify the best option, options that offer future potential with further practice or training, options that could be used for limited activities, and no-go options based on physical needs and abilities.

Beyond motor control, vision and hearing must be evaluated to fully characterize the user’s needs. Vision assessments are typically carried out by ophthalmologists, optometrists, or vision specialists and are crucial to understand the needs of the candidates to be able to provide visual accommodations (e.g., bigger text) when necessary. Similarly, evaluating auditory capabilities is necessary to determine whether AAC candidates are effectively receiving auditory input in their environment. Hearing assessments focus on candidate responses to spoken instructions, questions, responses, and feedback from text-to-speech outputs on AAC technologies.

Ultimately selecting an AAC method requires close liaison with motor control specialists (e.g., occupational and physiotherapists) to consider vision and hearing abilities alongside physical requirements such as posture and fine motor skills. Interdisciplinary AAC collaborations are imperative in ataxia, which can affect multiple sensorimotor domains. For instance, Friedreich’s ataxia is associated with partial vision and/or hearing loss as well as physical symptoms affecting motor control skills (e.g., peripheral neuropathy). Involving an interdisciplinary team ensures that the selected AAC intervention addresses any communication needs while also considering the interplay with other aspects of the user’s sensorimotor function.

It is important that communication partner dynamics and environmental supports are identified to develop an operational profile for intervention. This encompasses legislative or regulatory information that either supports or imposes restrictions on the utilization of AAC interventions for the candidate. Community attitudes—from family and co-workers to the broader public—should be identified so that opportunities for participation as well as their understanding of AAC and skills are known.

Pooled information from the multifaceted AAC assessment process (spanning case history, communication skills and needs, and sensorimotor capabilities) helps the team identify short- and long-term goals for intervention [31], which is the second area of focus of the Participation Model. Many times, trials with specific AAC modalities are important for the team to home in on the most effective intervention. It may be that different AAC techniques are better suited for some environments. For example, an SGD may be effective in a quiet setting at home, but not in a noisy restaurant. Proactive planning for future intervention is essential since the needs and skills of a person with ataxia change over time. In turn, this may alter their ability to use their current intervention [31].

## Eye tracking

Eye tracking technology can be useful where ataxia limits the use of traditional input devices such as a keyboard or mouse due to impaired coordination of movement. Eye-tracking technology can be effectively used as a mode of AAC in the context of ataxia (see Consumer Case Study 1). Eye-tracking devices can be used without requiring any physical touch, which is advantageous for people with ataxia where impaired movement can limit timing and accuracy of touch. Users can type out messages by looking at on-screen keyboards where the eye tracker can select keys based on where the user is looking, and the selection can be made by dwelling on a key for a certain period or by using blink or switch activation. Like other AAC modalities, this is often combined with predictive text and phrase selection to minimize the amount of typing required. Once a message is composed, eye-tracking AAC devices can use in conjunction with text-to-speech technology to verbalise the message.

Beyond communication, eye-tracking technology can also give users with ataxia greater control of their environment. This can include full control of a computer, enabling users to navigate the web, use software, and even create art or music, depending on the software’s capabilities. It can extend to operating household electronics, computers, and even wheelchairs, providing a greater degree of independence.

## Potential limitations of technological AAC solutions

There are multiple reasons why AAC options are not adopted or are discontinued by potential users [32] (see also see Consumer Case Study 2). Progressive neurological diseases lead to changes in abilities (Fig. 1). This means that AAC approaches need to be adaptable over time. Technology that works well at one stage may become less effective with degeneration. High-tech solutions like eye-tracking devices may be complex to set up and use, especially for individuals who are not technologically able or are experiencing cognitive decline. The learning requirements can be burdensome with some individuals finding it difficult or frustrating to use these technologies effectively [33]. Motor control and coordination issues common in ataxia, including spasticity can interfere with the use of assistive technologies [34]. In ataxia specifically, involuntary eye movements or difficulties with maintaining steady head and neck control can affect the accuracy of eye-tracking systems [35]. Advanced assistive technologies can be prohibitively expensive and may not be covered by insurance or healthcare systems. This can limit access to those who might benefit from them the most, especially as individuals with significant disability are often underemployed or unemployed [36]. The dynamic and individual nature of communication needs in progressive neurological disease mandates that AAC approaches need to be customised. There is often a significant need for individual customization of assistive devices, which can be time-consuming and requires professional expertise, making it less accessible for those in remote or under-resourced areas. Alongside the factors of cost and customisation, technology often presents with technical and maintenance issues. Dependence on technology means that technical failures can leave individuals without a means of communication or control over their environment. Similarly, regular maintenance and troubleshooting can be challenging for users and caregivers. AAC devices are often used in conjunction with other approaches, requiring integration and interaction between approaches. Technology may not always integrate seamlessly with other devices and services needed for daily living. Compatibility issues can restrict the functionality and efficiency of assistive devices. To optimise set up, access and continued use of approaches, adequate training for users and ongoing support is needed. Caregivers and family members also often benefit from training to assist effectively.

Personal factors outside of the technology or approach itself can alter a user’s experience. Fatigue is common in mitochondrial disorders and neurodegenerative diseases generally [37]. Using assistive technologies often requires sustained attention and concentration, which impacts access and an individual’s ability to use an approach for extended periods. This is related to the interaction with cognitive functioning, where some individuals with ataxia may experience cognitive decline, impacting their ability to effectively use or adapt to new technologies. Lastly, AAC approaches can present a social barrier where the use of assistive technology is stigmatized or misunderstood by others. This can lead to feelings of isolation or embarrassment for the user.

## Future directions

The future of AAC will likely incorporate brain computer interfaces (BCI), building on recent work examining non-invasive brain signal interpretation. There are some recent advancements that will be relevant for people with ataxia. Meta's work on decoding speech from non-invasive brain recordings was recently published in Nature Machine Intelligence [38]. Their work aimed to amalgamate neurological signals and artificial intelligence through magnetoencephalography and electroencephalography. Apple recently patented technology that detects bio-signals using dynamic selection of electrodes" [39] within AirPods. Effectively interpreting brain signals through the ear using sensors embedded within ear pods. Alongside this work there is a strong push for supporting voice of the patient through voice banking. This has gained traction in conditions like Amyotrophic Lateral Sclerosis where disease trajectory is steep, and perseveration of communication is a priority [40]. The role of voice banking in slowly progressive but ultimately fatal disorders like ataxia is less clear. Where disease onset is in childhood, voice banking with an immature voice for later use as an adult is undesirable. As disease worsens, your own ‘healthy’ voice deteriorates. The best way to integrate a stored voice in an AAC set up that caters to multiple physical restrictions incumbent in the condition is also unclear. Trials are underway to evaluate the utility of voice banking in progressive neurological disease. In a recent ground-breaking development, a paralysed woman in the US successfully communicated using a digital avatar controlled through a BCI [41]. This was achieved by using high-density surface recordings of the speech cortex in a clinical-trial participant presenting with severe limb and vocal paralysis. The aim was to achieve real-time decoding across three interconnected speech-related output modalities: text, speech audio, and facial-avatar animation, encompassing lip sync and emotional expressions. These are tangible examples of the fusion between brain-computer interfaces with voice banking and the advancement of language AI. This integration holds the potential to enable individuals to preserve their voice, even in instances where they have lost the ability to speak.

## Recommended approaches when investigating suitability and allocation of AAC for people with ataxia

People with a hereditary ataxia should all receive a comprehensive assessment of their communication. This could include a clinical speech examination accompanied by patient reported outcomes and establishment of baseline function. Following establishment of needs, and whether AAC would enhance communication outcomes, a thorough assessment by a speech-language pathologist and potentially an occupational therapist specialized in AAC could be conducted to determine the most suitable system (see Table 1 for AAC resources). The chosen system needs to be evaluated and accurately calibrated for the individual’s use. Systems can be customised according to each interface and the individual’s preferences and abilities, possibly adjusting the sensitivity and latency times. Adequate training and support are needed for all AAC users, caregivers, and communication partners.

To address these limitations, continuous support from healthcare providers, regular assessments, and updates to technology are required. Manufacturers and service providers must work towards creating more adaptable, user-friendly, and integrated solutions that can evolve with the progression of the disease and the changing needs of the individual. Additionally, advocacy for better insurance coverage and more widespread adoption can help make these technologies more accessible to those in need.

## Data Availability

Not applicable

## Appendix

**Table:**

**Consumer Use Case 1** **Commentary from Scott Blackman (48 years old. diagnosed at 12 years)** • I have Friedreich ataxia. This has changed my speech. • In 2017, I was intubated for an unrelated medical condition and this permanently damaged one of my vocal cords. • Deterioration due to Friedreich’s ataxia and the vocal impairment pushed me towards using my computer for communicating. • I was interested in trialling eye tracking to help writing emails and other work. I was told that eye-tracking may not work in FA because of the “eye problems”. • My computer science background and understanding of AI meant I was able to teach and train the system, learning different techniques and finding limitations (i.e., cut and paste is very involved and incredibly time-consuming). • I also learnt that glare from light impacts the ability of the lasers to interpret my instructions, positioning of the screen is important. • Corrective eyewear (I wear glasses), medication, alcohol, hydration level, sleep deprivation, pulled muscles (neck, shoulders or back), posture and physical activity can also affect eye tracking for me. • Most of the software trialled provided basic keyboard functionality, but they were less usefyl for complex tasks, like writing my Masters thesis. • Some systems have superior eyes tracking functionality but are accompanied by poor keyboard functionality. • The preferred trigger method (primary input) is Dwell. I can adjust Dwell Time according to eye fatigue throughout the day. Tracking on both eyes with Time out. I have tried the right eye and left eye, and both eyes with Dwell and Blink. • I use my eye-gaze for my university studies, which requires use of software programming software and artistic software like Photoshop, Illustrator, as well as the Microsoft Office Suite. All with varying degrees of success. Success has required extensive trial and error.	

**Table:**

**Consumer Use Case 2** **Commentary from Dr Peter Gibilisco (60 years old. Friedreich ataxia since 45 years)** As an individual with Friedreich’s Ataxia, I have been severely impacted by the limitations it places on my physical capabilities. The most notable and devastating damage has been done to my ability to communicate. For example, today I find it impossible to use my keyboard. The only way I can use my keyboard at all is with the help of specific academic support workers, who with training and experience, have learned to understand most of my speech. Even so, my speech dysarthria still hinders my ability to communicate with my support workers, and to dictate what I need to say in my academic or everyday writings. Currently, there are many options available to make communicating easier for someone like me. That being said, there are not many that are specifically catered to my individual needs. An example of this is Eye Gaze. Eye Gaze is a communication software that tracks the eye movements of the individual and allows them to type through an onscreen keyboard. For this, you have to be able to focus on the keys to spell out the words and make corrections accordingly. For someone like me, however, this will not work. My Nystagmus is very severe and causes my eyes to frequently move rapidly. It is the root cause of my blindness, and it will never allow me to focus on any keys on the screen. My dysarthric speech, severely limited vision through nystagmus and difficulty in hearing has also limited the possibility of assistance through medical aids. Friedreich’s Ataxia is a very rare and individualised disability, and as a result it doesn’t fit in with generalised medical theory. Because I depend heavily on my computer to communicate, I was advised to use a software known as Grid 3. It is a basic software that uses very simplistic movements to make it function, and the onscreen keyboard emits lights and sounds to indicate which letters or words you can click on. It uses a switch and sound cues which I found was helpful to me for some time. My main barriers to communication are Nystagmus and Dysarthria which are the main controlling objects of my disability and are therefore fundamental in the use of Friedreich’s Ataxia.	

## Declarations

**Ethical Approval**

not applicable

## References

[1] Poole ML, Brodtmann A, Darby D, et al. Motor Speech Phenotypes of Frontotemporal Dementia, Primary Progressive Aphasia, and Progressive Apraxia of Speech. J Speech, Language, Hearing Res. 2017:1-15. 10.1044/2016_JSLHR-S-16-0140 10.1044/2016_JSLHR-S-16-0140 28289749

[2] Noffs G, Perera T, Kolbe SC, et al. What speech can tell us: A systematic review of dysarthria characteristics in Multiple Sclerosis. Autoimmunity Rev. 2018;17(12):1202–9. 10.1016/j.autrev.2018.06.010. 30316992 10.1016/j.autrev.2018.06.010

[3] Magee M, Copland D, Vogel AP. Motor speech and non-motor language endophenotypes of Parkinson’s disease. Expert Rev Neurotherapeut. 2019;19(12):1191–200. 10.1080/14737175.2019.1649142. 10.1080/14737175.2019.1649142 31343928

[4] Chan JCS, Stout JC, Vogel AP. Speech in prodromal and symptomatic Huntington’s disease as a model of measuring onset and progression in dominantly inherited neurodegenerative diseases. Neurosci Biobehav Rev. 2019;107:450–60. 10.1016/j.neubiorev.2019.08.009. 31419452 10.1016/j.neubiorev.2019.08.009

[5] Gibilisco P, Vogel AP. Friedreich ataxia. BMJ 2013;347 10.1136/bmj.f7062 10.1136/bmj.f7062 24301267

[6] Vogel AP, Graf LH, Magee M, et al. Home-based biofeedback speech treatment improves dysarthria in repeat-expansion SCAs. Annals Clin Trans Neuro. 2022;n/a(n/a) 10.1002/acn3.51613 10.1002/acn3.51613 PMC9380135 35726838

[7] Venugopalan S TJ, Yang SJ, Seaver K, Cave RJ, Jiang PP, Zeghidour N, Heywood R, Green J, Brenner MP. Speech Intelligibility Classifiers from 550k Disordered Speech Samples. *IEEE International Conference on Acoustics, Speech and Signal Processing (ICASSP)* 2023:1-5.

[8] Folker JE, Murdoch BE, Rosen KM, et al. Differentiating profiles of speech impairments in Friedreich’s ataxia: a perceptual and instrumental approach. Int J Language Commun Disorders. 2012;47(1):65–76. 10.1111/j.1460-6984.2011.00078.x. 10.1111/j.1460-6984.2011.00078.x 22268902

[9] Vogel AP, Wardrop MI, Folker JE, et al. Voice in Friedreich Ataxia. J Voice. 2017;31(2):243.e9-43.e19. 10.1016/j.jvoice.2016.04.015. 27501923 10.1016/j.jvoice.2016.04.015

[10] Folker JE, Murdoch BE, Cahill LM, et al. Articulatory Kinematics in the Dysarthria Associated with Friedreich's Ataxia. Motor Control 2011;15(3):376-89. [published Online First: 2011/09/01] 10.1123/mcj.15.3.376 21878690

[11] Folker JE, Murdoch BE, Cahill LM, et al. Kinematic analysis of lingual movements during consonant productions in dysarthric speakers with Friedreich’s ataxia: A case-by-case analysis. Clin Linguist Phonetics. 2011;25(1):66–79. 10.3109/02699206.2010.511760. 10.3109/02699206.2010.511760 20932172

[12] Poole ML, Wee JS, Folker JE, et al. Nasality in Friedreich ataxia. Clin Linguist Phonetics. 2015;29(1):46–58. 10.3109/02699206.2014.954734. 10.3109/02699206.2014.954734 25207996

[13] Folker JE, Murdoch BE, Cahill LM, et al. Dysarthria in Friedreich’s ataxia: a perceptual analysis. Folia Phoniatrica et Logopaedia. 2010;62(3):97–103. 10.1159/000287207 20424464

[14] Vogel AP, Maruff P, Reece H, et al. Clinically meaningful metrics of speech in neurodegenerative disease: Quantification of speech intelligibility and naturalness in ataxia. *medRxiv* 2023:2023.03.28.23287878. 10.1101/2023.03.28.23287878

[15] Rance G, Corben L, Barker E, et al. Auditory Perception in Individuals with Friedreich’s Ataxia. Audio Neuro. 2010;15(4):229–40. 10.1159/000255341 19893304

[16] Seyer LA, Galetta K, Wilson J, et al. Analysis of the visual system in Friedreich ataxia. J Neurol. 2013;260(9):2362-9. 10.1007/s00415-013-6978-z [published Online First: 20130618] 10.1007/s00415-013-6978-z 23775342

[17] Menegoni F, Milano E, Trotti C, et al. Quantitative evaluation of functional limitation of upper limb movements in subjects affected by ataxia. Eur J Neurol. 2009;16(2):232–9. 10.1111/j.1468-1331.2008.02396.x. 19146643 10.1111/j.1468-1331.2008.02396.x

[18] Veruggio G, Panella M. Cerebral Palsy: Springer.

[19] Elsahar Y, Hu S, Bouazza-Marouf K, et al. Augmentative and Alternative Communication (AAC) Advances: A Review of Configurations for Individuals with a Speech Disability. Sensors (Basel) 2019;19(8) 10.3390/s19081911 [published Online First: 20190422] 10.3390/s19081911 PMC6515262 31013673

[20] Beukelman DR, Light J. Augmentative and alternative communication: Supporting children and adults with complex communication needs: Brookes Publishing 2020.

[21] Creer S, Enderby P, Judge S, et al. Prevalence of people who could benefit from augmentative and alternative communication (AAC) in the UK: determining the need. Int J Lang Commun Disord 2016;51(6):639-53. 10.1111/1460-6984.12235 [published Online First: 20160426] 10.1111/1460-6984.12235 27113569

[22] Vogel AP, Sobanska A, Gupta A, et al. Quantitative Speech Assessment in Ataxia-Consensus Recommendations by the Ataxia Global Initiative Working Group on Digital-Motor Markers. *Cerebellum* 2023 10.1007/s12311-023-01623-4 [published Online First: 20231028] 10.1007/s12311-023-01623-4 PMC11102369 37897626

[23] Dietz A, Quach W, Lund SK, et al. AAC assessment and clinical-decision making: the impact of experience. Augment Altern Commun. 2012;28(3):148–59. 10.3109/07434618.2012.704521. 22946990 10.3109/07434618.2012.704521

[24] Bailey RL, Stoner JB, Parette Jr HP, et al. AAC team perceptions: Augmentative and alternative communication device use. Educ Train Develop Disabilities 2006:139-54.

[25] Moorcroft A, Scarinci N, Meyer C. Speech pathologist perspectives on the acceptance versus rejection or abandonment of AAC systems for children with complex communication needs. Augment Altern Commun. 2019;35(3):193-204. 10.1080/07434618.2019.1609577 [published Online First: 20190715] 10.1080/07434618.2019.1609577 31307237

[26] ASHA. Augmentative and Alternative Communication American Speech and Hearing Association2023 [Available from: https://www.asha.org/practice-portal/professional-issues/augmentative-and-alternative-communication/ Accessed 17 November 2023 2023.

[27] Treadwell TW, Leach E, Stein S. The Social Networks Inventory: A Diagnostic Instrument Measuring Interpersonal Relationships. Small Group Res. 1993;24(2):155–78. 10.1177/1046496493242001.

[28] Swinburn K, Porter G, Howard D. CAT: Comprehensive Aphasia Test. Hove, UK: Psychology Press; 2004.

[29] Dunn DM. Peabody picture vocabulary test fifth edition (PPVT-5). *Minneapolis, MN.*, 2018.

[30] Switch Assessment and Planning Framework for Individuals with Physical Disabilities: AceCentre; [Available from: https://acecentre.org.uk/resources/switch-assessment-planning-framework-individuals-physical-disabilities Accessed 5th January 2024.

[31] Forest M, Pearpoint J, O'Brien J. MAPS. Educational Psych Prac. 1996;11(4):35-40. 10.1080/0266736960110407

[32] Johnson JM, Inglebret E, Jones C, et al. Perspectives of speech language pathologists regarding success versus abandonment of AAC. Augment Altern Commun. 2006;22(2):85–99. 10.1080/07434610500483588. 17114167 10.1080/07434610500483588

[33] Iacono T, Lyon K, Johnson H, et al. Experiences of adults with complex communication needs receiving and using low tech AAC: an Australian context. Disabil Rehabil Assist Technol. 2013;8(5):392–401. 10.3109/17483107.2013.769122[publishedOnlineFirst:20130423]. 23992458 10.3109/17483107.2013.769122

[34] Corben LA, Yiu EM, Tai G, et al. Probing the multifactorial source of hand dysfunction in Friedreich ataxia. J Clin Neurosci. 2019;64:71–6. 10.1016/j.jocn.2019.04.009[publishedOnlineFirst:20190422]. 31023572 10.1016/j.jocn.2019.04.009

[35] Stephen CD, Schmahmann JD. Eye Movement Abnormalities Are Ubiquitous in the Spinocerebellar Ataxias. Cerebellum. 2019;18(6):1130–6. 10.1007/s12311-019-01044-2. 31175630 10.1007/s12311-019-01044-2

[36] ABS. Australians Living with Communication Disability: Australia Bureau of Statistics, 2017.

[37] Penner IK, Paul F. Fatigue as a symptom or comorbidity of neurological diseases. Nat Rev Neurol. 2017;13(11):662-75. 10.1038/nrneurol.2017.117 [published Online First: 20171013] 10.1038/nrneurol.2017.117 29027539

[38] Défossez A, Caucheteux C, Rapin J, et al. Decoding speech perception from non-invasive brain recordings. Nature Machine Intel. 2023;5:1097–107. 10.1038/s42256-023-00714-5.

[39] Azemi E, Moin A, Pragada A, et al. 2023. USA.

[40] Mills T, Bunnell HT, Patel R. Towards personalized speech synthesis for augmentative and alternative communication. *Augment Altern Commun* 2014;30(3):226-36. 10.3109/07434618.2014.924026 [published Online First: 20140715] 10.3109/07434618.2014.924026 25025818

[41] Metzger SL, Littlejohn KT, Silva AB, et al. A high-performance neuroprosthesis for speech decoding and avatar control. Nature 2023;620(7976):1037-46. 10.1038/s41586-023-06443-4 [published Online First: 20230823] 10.1038/s41586-023-06443-4 PMC10826467 37612505

